# Evaluating the impact of Arteriovenous Fistula (AVF) creation on kidney function

**DOI:** 10.1007/s40620-025-02316-3

**Published:** 2025-06-13

**Authors:** Sri Ram Ramasamy Muthuraman, Emma Aitken

**Affiliations:** 1 University of Glasgow, Glasgow, UK; 2 Department of Renal Surgery, Queen Elizabeth University Hospital, Glasgow, UK

**Keywords:** Arteriovenous fistula, Pre-dialysis, Kidney failure

## Abstract

**Background:**

Arteriovenous fistula (AVF) creation may slow down the decline of kidney function as shown in few previous studies. Our aim was to evaluate the effect of AVF creation on kidney function and dialysis initiation in predialysis patients.

**Methods:**

Predialysis patients with AVFs and estimated glomerular filtration rate (eGFR) recorded on day of AVF creation and at 12 and 6 months pre- and post-AVF creation were identified. Rate of decline (RoD) of eGFR and Kidney Failure Risk Equation Score (KFRE) was recorded pre- and post-AVF creation at 6 and 12 months. Patients with non-functioning AVFs were identified and RoD was compared to functioning AVFs. Patients undergoing haemodialysis pre- and post-AVF creation were identified.

**Results:**

Overall, 368 patients and 435 patients were identified for the 12-month and 6-month group, respectively. Rate of decline of eGFR 6 months pre-AVF creation was -0.33 ml/min/1.73m2/month which slowed down to -0.23 ml/min/1.73m2/month in the 6 months post-AVF creation. Rate of decline of eGFR 12 months pre-AVF creation was -0.37 ml/min/1.73m2/month and slowed down to -0.19 ml/min/1.73m2/month in the 12 months post-AVF creation. KFRE increased at a slower rate at 6 and 12 months post-AVF. One hundred sixty-two patients had functioning AVFs while 29 patients had non-functioning AVFs at 6 months post-AVF creation. Rate of decline of eGFR in patients with functioning AVF was -0.22 ml/min/1.73m2/month compared to -0.32 ml/min/1.73m2/month in patients with non-functioning AVF; 246 patients (62.3%) were commenced on haemodialysis in the 12 months pre- and post- AVF creation.

**Conclusion:**

AVF creation was associated with a slower rate of decline of kidney function. However, this is unlikely to delay the commencement of haemodialysis in predialysis patients.

**Graphical abstract:**

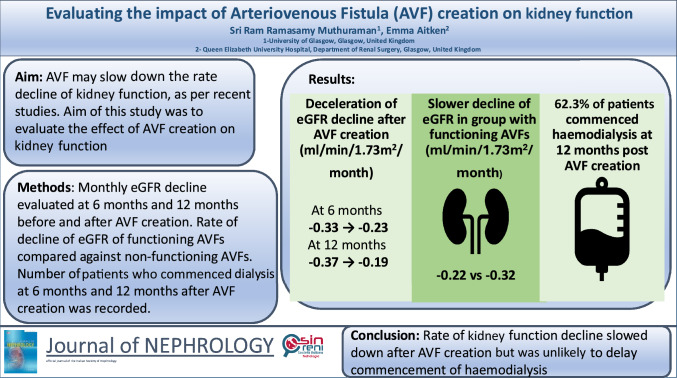

## Introduction

Kidney failure imposes a significant disease burden on patients [1] and a significant cost for healthcare systems worldwide [2]. Within the UK, in 2021, most patients with kidney failure were commenced on haemodialysis as kidney replacement therapy (KRT) [3]. Haemodialysis requires vascular access. Arteriovenous fistulae (AVFs) remain the preferred form of vascular access with a lower frequency of future infections and further interventions to ensure patent vascular access when compared to alternative forms of vascular access [4].

Kidney function naturally declines over time with age-related decline in Glomerular Filtration Rate (GFR) being approximately 1 ml/min/1.73 m^2^ per year [5]. However, this rate of decline can be markedly increased in chronic kidney disease (CKD), resulting in more rapid progression to kidney failure [6]. Some factors that may increase the decline of estimated GFR (eGFR) and hasten the progression of CKD to kidney failure include hypertension, poor glycaemic control in diabetes, proteinuria and smoking [7]. As the definition of kidney failure is based on eGFR, the decline of kidney function in patients with CKD allows us to predict the risk of kidney failure, with a faster decline associated with an increased risk of kidney failure [8].

The Kidney Failure Risk Equation (KFRE) score can be used to estimate the probability of an individual requiring KRT within the next 5 years. It is a well-validated formula based on a patient’s age, sex, eGFR and urine Albumin:Creatinine Ratio (uACR) [9, 10]. An annual KFRE score of greater than 20% has been shown to be the optimal threshold to initiate referral for AVF creation [11]. Hence, it is important to understand the rate of decline of eGFR and kidney function, as this gives us insight into CKD progression and allows us to plan for KRT in patients who are fast approaching kidney failure.

Several retrospective cohort studies have shown that the rate of decline dropped after the creation of AVFs [12–15]. This suggests that there could be a possible role for AVF creation in delaying the progression of CKD and that it might serve as a potential kidney-preserving therapy in patients with advanced CKD. However, previous studies looking at the possible effects of AVF creation on eGFR decline struggled to find an appropriate control group that controlled for confounding variables [12, 13]. Our study overcomes this limitation by comparing the rate of decline in predialysis patients with mature AVFs against predialysis patients whose AVFs were non-functioning.

Our study aims to evaluate the effects of AVF creation on the rate of decline of kidney function in the predialysis patient cohort. We hypothesise that AVF creation will result in a slower decline in eGFR and that this would delay the need for haemodialysis in predialysis patients. Our study is novel in that it measures the effect of AVF creation on the 5-year risk of KRT and identifies the proportion of patients who start haemodialysis 6 and 12 months after AVF creation. These outcomes would help identify whether AVF creation might be able to delay the initiation of KRT.

## Methods

### Study design

This is a single centre, retrospective cohort study of all patients attending our predialysis care clinic at the Queen Elizabeth University Hospital, Glasgow for arteriovenous fistula creation between 1st January 2020 and 31st December 2023.

### Population

**Fig. 1:**
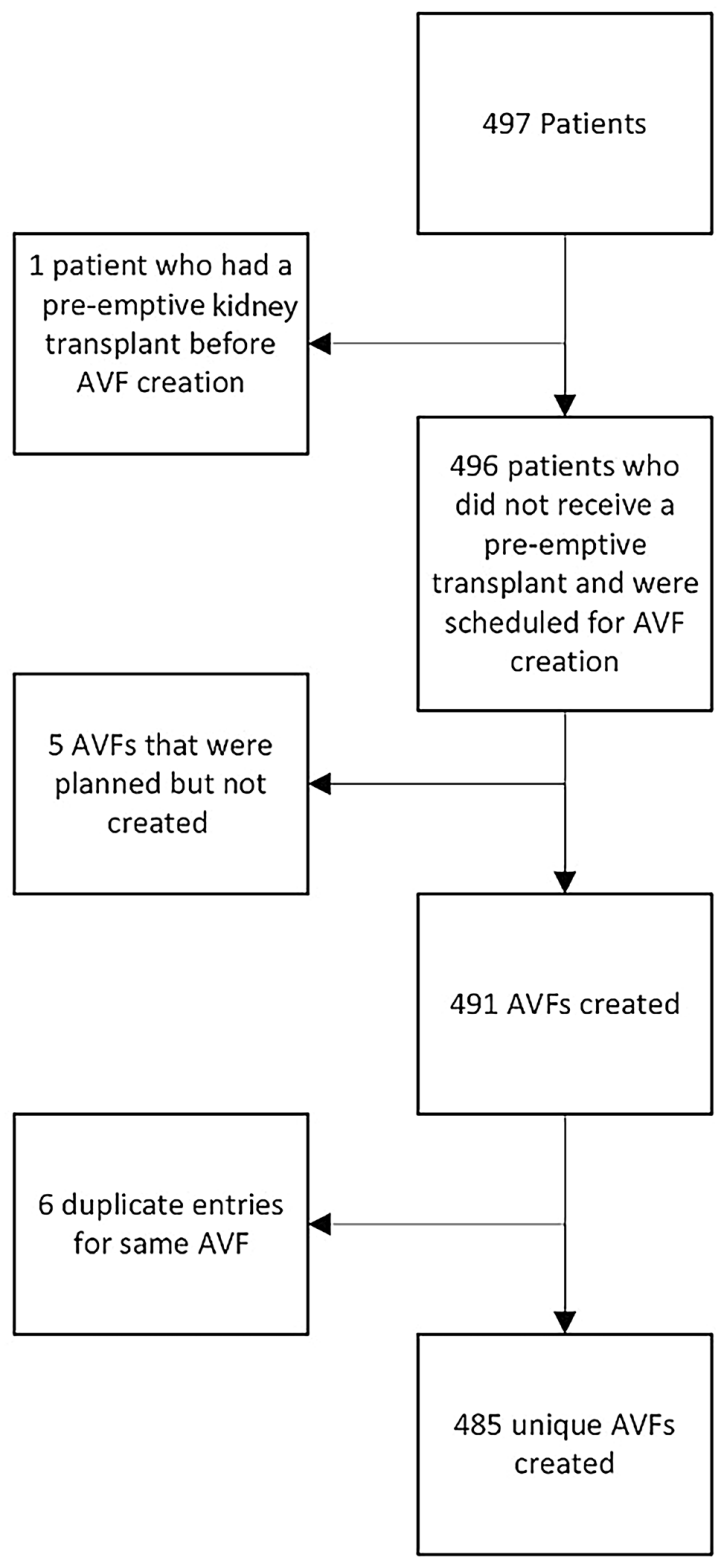
Study population of consecutive unique AVFs created from 2020 to 2023

Patients were included if they were aged 18 years or older, had not yet commenced dialysis and had an AVF created during the study period (*n* = 497). Patients undergoing pre-emptive transplantation were excluded if transplantation occurred before AVF creation (*n* = 1). Patients who had multiple AVFs in the time period, or duplicate entries within our database were also excluded (*n* = 11). The final cohort of 485 unique AVFs were then studied at 6 months prior to, and post, AVF creation or 12 months prior to, and post, AVF creation (Fig. 1).

For the cohort that was studied 12 months pre and post AVF creation, patients were included if they had recorded eGFR values for 12 months pre and post AVF creation. Hence, patients that were unknown to the renal service 12 months prior to AVF creation and patients with no recorded eGFR 12 months after AVF creation were excluded (*n* = 436). Patients that were transplanted in the 12 months post AVF creation were also excluded (*n* = 411). Forty-three patients had died between AVF creation and 12 months post AVF creation and were excluded (*n* = 368).

Regarding the cohort that was studied 6 months pre and post AVF creation, one patient had an AVF created for an AKI that later recovered. This anomalous result was excluded (*n* = 484). Patients were then included if they had recorded eGFR values for 6 months pre and post AVF creation. Therefore, patients unknown to the renal service 6 months prior to AVF creation and patients with no recorded eGFR 6 months after AVF creation were excluded (*n* = 462). Patients that were transplanted in the 6 months post AVF creation were also excluded (*n* = 454). Nineteen patients died in the 6 months post AVF creation and were excluded (*n* = 435).

### Data collection

Data were extracted from the Scottish Electronic Renal Patient Record (SERPR) database. Kidney function was recorded as eGFR calculated using the modified Chronic Kidney Disease Epidemiology Collaboration (CKD-EPI) formula [16]. Demographic data including site of AVF, age, sex, ethnicity, primary kidney disease, eGFR and presence of co-morbidities such as diabetes and hypertension were identified at the time of AVF creation. Patients were included for analysis at 6- and 12-month time points if they were alive, had not been transplanted and eGFR data were available ± 28 days from the anticipated date of follow-up (n = 368 at 12 months; *n* = 435 at 6 months). Patients with available urinary ACR ± 28 days also had a 5-year KFRE score calculated using the 4-variable 5-year KFRE formula [9, 10].

All AVFs were classified as functioning or non-functioning based on their clinical appearance as assessed by an experienced vascular access nurse 6 weeks post-AVF creation. Functioning AVFs were defined as AVFs that had palpable thrill and/or audible bruit on auscultation whether or not they were deemed sufficiently mature to sustain dialysis yet.

eGFR was recorded 6 and 12 months prior to AVF creation (± 28 days), on the day of AVF creation and 6 and 12 months after AVF creation (± 28 days) for pre-dialysis patients (or dialysis status in those who had started dialysis). End of patient follow-up occurred at commencement of haemodialysis, transplantation, end of follow-up period (either 6 or 12 months) or death, whichever occurred first.

### Outcomes

The primary outcome was the rate of decline of kidney function after AVF creation in the functioning AVF group compared to the non-functioning AVF group. The secondary outcomes include the rate of decline of kidney function before and after AVF creation for all AVFs, rate of decline of 5-year KFRE before and after AVF creation for all AVFs and the proportion of patients who were started on haemodialysis prior to AVF creation compared to the proportion of patients who were started on haemodialysis after AVF creation within the study period.

### Statistical analyses

The monthly decline in eGFR was calculated by dividing the difference in recorded eGFR values at 12- or 6-month intervals by the number of months between the recorded eGFR values. The decline in 5-year KFRE was calculated in a similar manner. Data are presented as total number and percentage for categorical variables and as mean ± standard deviation or median and interquartile ranges for continuous variables. Continuous variables were compared using t tests.

The rate of eGFR decline per month and rate of decline of 5-year KFRE per month after AVF creation in patients with a functioning AVF and patients with a non-functioning AVF were compared using paired t-test.

The within-patient monthly eGFR decline and 5-year KFRE pre and post AVF creation was also compared using paired t-test.

The rate of decline of kidney function in the 6 months following AVF creation was confirmed to be normally distributed. Univariate logistic regression was performed with baseline demographics (age, gender, ethnicity, primary renal diagnosis, presence/absence of diabetes, presence/absence of hypertension, presence/absence of early thrombosis) as exploratory variables, and rate of decline of kidney function in the 6 months post-AVF creation as the outcome variable. The significance threshold for inclusion in further multiple regression models was set at *p* < 0.1. An appropriate multiple regression model was then produced including variables which met the significance threshold in a stepwise fashion.

### Research ethics approval

No formal research ethical approval was required for this retrospective project in line with the Health Research Authority decision tool. The project was however recorded with the local renal audit and research database, and necessary approvals from the Caldicott Guardian were obtained.

## Results

### Demographics

Four hundred eighty-five patients were identified that fit the inclusion criteria. Baseline characteristics are shown in Table 1. The median age was 65 years and 61.2% of patients were male. Among participants, 61.4% were White Scottish and 34.8% had a primary renal diagnosis of diabetic nephropathy; 50.7% of patients had diabetes mellitus and 53.6% of patients had hypertension. The median (IQR) renal function at time of AVF creation was 10.5 (8.2, 12.9) ml/min/1.73 m^2^. 5-year KFRE was calculated for 36 patients and the median (IQR) 5-year KFRE on the day of AVF creation was 0.72 (0.5, 0.9). Overall, 29.69% of patients were on haemodialysis at time of AVF creation.

**Table 1:** Baseline characteristics of included patients

Variable	*n* = 485
**Age** (years), median (IQR)	65 (55,74)
**Sex** (male), *n* (%)	297 (61.2)
**Race**, *n* (%)	
African	14 (2.9)
Asian- Indian	7 (1.4)
Asian other	3 (0.6)
Bangladeshi	1 (0.2)
Chinese	4 (0.8)
Pakistani	18 (3.7)
White Scottish	298 (61.4)
Unknown	140 (28.9)
**Primary Renal Diagnosis**, *n* (%)	
Diabetic nephropathy	169 (34.9)
Familial/hereditary nephropathy	37 (7.6)
Glomerular disease	65 (13.4)
Hypotension/renovascular disease	62 (12.8)
Other systemic disease affecting the kidneys	31 (6.4)
Tubulointerstitial nephritis	6 (1.2)
Miscellaneous	115 (23.7)
**Co-morbidities**, *n* (%)	
Diabetes	246 (50.7)
Hypertension	260 (53.6)
eGFR at time of AVF creation (ml/min/1.73 m^2^), median (IQR)	10.5 (8.2, 12.9)
5-year KFRE at time of AVF creation, median (IQR)	0.72 (0.5, 0.9)
Haemodialysis at time of AVF creation, *n* (%)	144 (29.7)

### Rate of decline for all AVFs at 6 months and 12 months

Median (Interquartile range [IQR]) rate of decline at 6 months pre-AVF creation was − 0.33 (− 0.63, − 0.10) ml/min/1.73 m^2^/month. The median (IQR) rate of decline 6 months post-AVF creation was −0.23 (− 0.43, − 0.017) ml/min/1.73 m^2^/month.

**Fig. 2:**
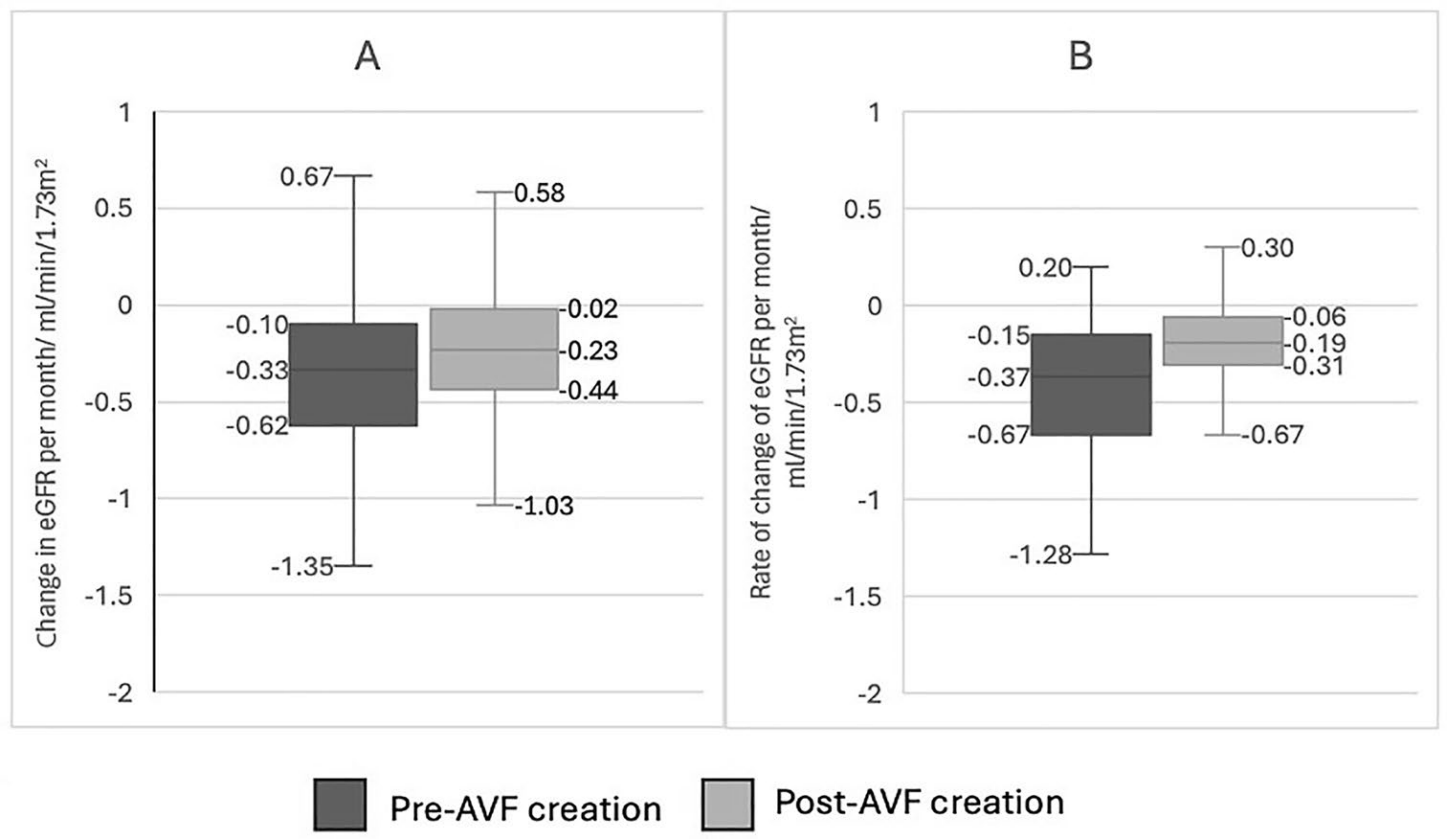
Rate of change of eGFR per month in patients 6 months (**A**) and 12 months (**B**) before and after AVF formation

The median (IQR) rate of decline 12 months pre-AVF creation was − 0.37 (− 0.66, − 0.16) ml/min/1.73 m^2^/month. The median (IQR) rate of decline 12 months post-AVF creation was − 0.19 (− 0.31, − 0.067) ml/min/1.73 m^2^/month. These results are illustrated in Fig. 2.

The decrease in rate of decline after AVF creation at 6 and 12 months was statistically significant (6 months: *p* < 0.0001; 12 months: *p* < 0.000001).

### Rate of increase of 5-year KFRE for all AVFs at 6 months and 12 months

The KFRE was calculated for 35 patients in the 6-month group. The median (IQR) rate of 5-year KFRE score increase pre-AVF creation was 0.012 (0.0049, 0.021) per month and the median (IQR) rate of 5-year KFRE score increase post-AVF creation was 0.0048 (− 0.00042, 0.012) per month.

Five-year KFRE was calculated for 14 patients in the 12-month group. The median rate of increase of 5-year KFRE pre-AVF creation was 0.017 (0.0082, 0.024) per month and the median rate of increase of 5-year KFRE post-AVF creation was 0.0037 (− 0.00038, 0.0097). The reduction in the rate of increase of 5-year KFRE after AVF creation at 6 and 12 months was statistically significant (6 months: *p* = 0.019; 12 months: *p* = 0.0036).

### Rate of decline post AVF creation in functioning vs non-functioning at 6 months and 12 months

Six months after AVF creation, 162 patients had a functioning AVF while 29 had non-functioning AVFs.The median (IQR) rate of decline post-AVF creation was − 0.22 (− 0.42, − 0.03) ml/min/1.73 m^2^/month in the cohort with functioning AVFs and − 0.32 (− 0.48, − 0.017) ml/min/1.73 m^2^/month in the cohort with non-functioning AVFs. This difference was statistically significant (*p* = 0.025).

Twelve months after AVF creation, 89 patients had a functioning AVF while 18 had non-functioning AVFs. The median (IQR) rate of decline post-AVF creation was − 0.2 (− 0.38, − 0.058) ml/min/1.73 m^2^/month in the cohort with functioning AVFs and − 0.16 (− 0.23, − 0.083) ml/min/1.73 m^2^/month in the cohort with non-functioning AVFs. However, this was not statistically significant. (*p* = 0.33).

### Commencing haemodialysis pre AVF creation vs post AVF creation at 6 months and 12 months

Four hundred and sixty three and 438 patients were identified in the renal service at 6 months and 12 months prior to AVF creation, respectively.

At 6 months prior to their AVF creation, 390 patients were not on haemodialysis, 58 (14.9%) of these patients started on haemodialysis in the 6 months preceding AVF creation and 126 patients (32.3%) started on haemodialysis in the 6 months after AVF creation.

At 12 months prior to AVF creation, 395 patients were not on haemodialysis, 82 (20.8%) of these patients started on haemodialysis in the 12 months preceding AVF creation and 164 (41.5%) patients started on haemodialysis in the 12 months after AVF creation.

### Regression model at 6 months post AVF creation

The following variables met the significance threshold of p < 0.1 and were included in the multiple regression model: age, diabetes, rate of decline of kidney function in the 6 months prior to AVF creation. Notably early thrombosis was not found to be a statistically significant predictor on univariate analysis (*p* = 0.32).

The multiple regression model is outlined in Table 2. The R-squared value for the model is 0.63 (F statistic 23.4; *p* < 0.0001).

**Table 2:** Multiple regression model

	Unstandardised coefficients		*P*-value
	Β	S.E.	
Constant	0.04	0.03	
Age	− 0.12	0.02	< 0.001
Diabetes	0.64	0.54	< 0.001
Rate of decline in function in the 6 months prior to AVF	0.05	0.03	< 0.01

## Discussion

In this single-centre retrospective cohort study, we found that the creation of AVFs reduced the rate of decline in kidney function. When patients with functioning AVFs were compared to those with non-functioning AVFs, the group with functioning AVFs experienced a slower rate of decline after AVF creation. We also found that most predialysis patients commenced haemodialysis in the 12 months post-AVF creation despite the slower rate of decline and the slower increase in their 5-year KFRE score.

The improvement in the rate of decline in patients with kidney failure after the creation of AVFs that we observed was in line with the findings of multiple retrospective cohort studies. Initial studies by Golper et al. on this subject demonstrated improvements in rate of decline after AVF creation [15]. However, this study did not have a control group and used the less reliable 4-variable Modification of Diet in Renal Disease (MDRD) equation that included race as one of the variables when calculating eGFR [17]. Subsequent studies on the impact of AVF creation on kidney function by Dupuis et al. and Sumida et al. reported similar results [12, 14]. These studies involved predialysis patients who were undergoing peritoneal dialysis and predialysis patients who did not have AVFs as control groups, respectively. However, the patients on peritoneal dialysis had fewer co-morbidities and a lower median body mass index (BMI) than patients who had an AVF created [12], and the predialysis patients who did not have AVFs created had a higher prevalence of cardiovascular disease, liver disease and malignancy [14]. Hence, the control groups in these studies were less appropriate. Our study overcame this by having patients who had undergone AVF creation which had then failed to function as our control group. Since these patients were deemed suitable for AVF creation surgery after a pre-operative assessment that accounted for their co-morbidities [18], we can conclude that there was a similar level of baseline health in both the control and intervention groups of our study.

These studies only recorded the rate of decline after AVF creation, which might have limited application to clinical practice. Our study found that while AVF creation may improve the rate of decline and cause a slowing down in the 5-year KFRE in predialysis patients, most of these patients commence haemodialysis in the 12-month period following AVF creation. As such, while AVF creation in the predialysis patient population may delay the initiation of haemodialysis, this is unlikely to be by more than a year post-operatively, thus planning and allocation of resources for the initiation of haemodialysis should still be considered for these patients.

The deceleration of rate of decline following the creation of AVF could be due to the change in renal perfusion and haemodynamics [19]. The arteriovenous fistula creates a relatively low-resistance pathway for blood and this could result in a reduced cardiac afterload [20]. Moreover, as blood flows at a faster rate from arteries directly into the venous system via an AVF, blood is returned to the right atrium and ventricle at a faster rate, resulting in increased cardiac preload. This results in increased left ventricular ejection fraction and increased cardiac output [20–22] causing more oxygenated blood to perfuse the kidneys upon the creation of an AVF. As eGFR is inversely proportional to serum creatinine, which is now being cleared at a faster rate through the kidneys due to the increased renal blood flow, the creation of an AVF is associated with a deceleration in the rate of increase of serum creatinine values due to underlying CKD and hence results in a deceleration in the rate of decline in predialysis patients with CKD [12–15].

Another possible explanation for the deceleration of the rate of decline could be due to remote ischaemic preconditioning [12–15]. During the creation of an AVF, there is a subclinical steal phenomenon, resulting in relative ischaemia to a distal portion of a limb [12–15, 23, 24]. This ischaemia can result in a neurohormonal and systemic response which suppresses inflammation and apoptosis [24, 25]. Damage-associated molecular patterns (DAMPs) are released as part of this systemic response, some of which interact with renal tubular epithelial cells to reduce their turnover [24, 25]. DAMPs can also pre-condition the renal epithelial cell to dampen any cellular response to subsequent inflammatory or ischaemic events, which are often the underlying cause of the CKD [24, 25]. This results in the preservation of kidney function, as the renal epithelial cells are less likely to atrophy due to the underlying cause of CKD [26].

Most of the patients in our study commenced haemodialysis within the 12 months following their AVF creation surgery. This is indicative of a progression of their CKD and is likely because predialysis patients for whom AVF creation is indicated already have a lower baseline kidney function [6]. Following AVF creation, kidney function continues to decline, albeit at a slower rate, as the underlying cause of CKD is not treated by the creation of AVF. Hence, while the creation of AVFs may delay the initiation of dialysis in predialysis patients, it is unlikely to prevent this altogether for most patients.

Our study was limited to a single centre and therefore, the results of our study may have limited applicability. However, multiple studies from different centres have reported similar results to our study, which suggests that the findings of our study may be applicable to the wider predialysis patient population worldwide. Our study also relied on observational data and hence, could only identify a correlation between the creation of AVF and the deceleration of rate of decline.

In conclusion, our study found that patients experienced a slower decline in their eGFR and slower increase in their KFRE after AVF creation at both 6 and 12 months. We identified that most patients who undergo AVF creation commence haemodialysis in the 12 months after AVF creation. We also found that patients with functioning AVFs experienced a slower decline in their eGFR compared to patients with non-functioning AVFs at both 6 and 12 months. However, multivariate analysis at 6 months showed that the functional status of AVF was not a predictor of the rate of decline of eGFR. Our study was limited to observational data and hence, a further prospective study should be conducted to ascertain the clinical impact of AVF creation on kidney function and haemodialysis initiation.

## Data Availability

The dataset analysed during the current study is not publicly available to preserve the confidentiality of individuals in the study. Data may be shared on reasonable request to the corresponding author with permission of NHS Greater Glasgow and Clyde.

## Abbreviations

AVF: Arteriovenous Fistula
KRT: Kidney Replacement Therapy
eGFR: estimated Glomerular Filtration Rate
CKD: Chronic Kidney Disease
KFRE: Kidney Failure Risk Equation
IQR: interquartile range
